# Retraction Note: A candidate Chinese medicine preparation-Fructus Viticis Total Flavonoids inhibits stem-like characteristics of lung cancer stem-like cells

**DOI:** 10.1186/s12906-022-03569-2

**Published:** 2022-03-25

**Authors:** Xiaocheng Cao, Hui Zou, Jianguo Cao, Yinghong Cui, Shuwen Sun, Kaiqun Ren, Zhenwei Song, Duo Li, Meifang Quan

**Affiliations:** 1 College of Life Science, Hunan Normal University, Changsha, China; 2 Laboratory of Medicine Engineering, College of Medicine, Hunan Normal University, Changsha, China

**Retraction Note: BMC Complement Altern Med 16, 364 (2016)**

**https://doi.org/10.1186/s12906-016-1341-4**

The Editor has retracted this article. After publication, concerns were raised about three of the figures. Specifically:

- In Figure 5b two panels 1.0 and 2.0 shown at different magnifications appear to overlap
- In Figure 2b panel 8d appears to overlap with the Vector panel in Figure 6H of [1]
- In Figure 2d the PC panel appears to overlap with panels 1.0 and 2.0 in Figure 5b, and the SFC panel appears to overlap with the 0 panel of Figure 5b
- The PC panel in Figure 2d and the 1.0 and 2.0 panels in Figure 5b also appear to overlap with the 100 nM SiTF panel in Figure 9 of [2]

The Editor therefore no longer has confidence in the data presented. Xiaocheng Cao, Hui Zou, Jianguo Cao, Yinghong Cui, Shuwen Sun, Kaiqun Ren, Duo Li and Meifeng Quan agree with this retraction. Zhenwei Song has not responded to correspondence from the Editor about this retraction.
